# Prognostic Evaluation of DNA Index in HIV-HPV Co-Infected Women Cervical Samples Attending in Reference Centers for HIV-AIDS in Recife

**DOI:** 10.1371/journal.pone.0104801

**Published:** 2014-08-21

**Authors:** Albert Eduardo Silva Martins, Norma Lucena-Silva, Renan Gomes Garcia, Stefan Welkovic, Aureliana Barbosa, Maria Luiza Bezerra Menezes, Terezinha Tenório, Magda Maruza, Ricardo A. A. Ximenes

**Affiliations:** 1 Laboratório de imunogenética, Centro de pesquisas Aggeu Magalhães, Fundação Oswaldo Cruz, Recife, Brasil; 2 Laboratório de biologia molecular, Departamento de oncologia pediátrica, Hospital IMIP, Recife, Brasil; 3 Departamento Materno-Infantil e Centro Integrado de Saúde Amaury de Medeiro (CISAM-UPE), Universidade de Pernambuco, Recife, Brasil; 4 Departamento Materno-Infantil, Universidade Federal de Pernambuco, Recife, Brasil; 5 Hospital Correia Picanço, Secretaria de Saúde de Pernambuco, Recife, Brasil; 6 Departamento de Medicina Tropical, Universidade Federal de Pernambuco (UFPE), Recife, Brasil; University of Alabama at Birmingham, United States of America

## Abstract

**Introduction:**

Persistence of cervical infection caused by human papillomavirus (HPV) types with high oncogenic risk may lead to cervical intraepithelial neoplasia (CIN). The aim of the present study was to evaluate whether, in HIV-positive women, the presence of aneuploidy in cervical cell samples is associated with presence and evolution of CIN.

**Methods:**

The present study had two stages. In the first stage, comprising a cross-sectional study, the association between the presence of aneuploidy seen via flow cytometry and sociodemographic characteristics, habits and characteristics relating to HPV and HIV infection was analyzed. In the second stage, comprising a cohort study, it was investigated whether aneuploidy was predictive of CIN evolution.

**Results:**

No association was observed between the presence of aneuploidy and HPV infection, or between its presence and alterations seen in oncotic cytological analysis. On the other hand, aneuploidy was associated with the presence of CIN (p = 0.030) in histological analysis and with nonuse of antiretroviral therapy (p = 0.001). Most of the HIV-positive women (234/272) presented normal CD4+ T lymphocyte counts (greater than 350 cells/mm^3^) and showed a greater aneuploidy regression rate (77.5%) than a progression rate (23.9%) over a follow-up of up to two years.

**Conclusion:**

Although there was an association between the presence of cervical tissue lesions and the DNA index, the latter was not predictive of progression of the cervical lesion. This suggests that progression of the cervical lesion to cancer in HIV-positive women may also be changed through improvement of the immunological state enabled by using antiretroviral therapy.

## Introduction

Persistence of cervical infection caused by the human papillomavirus (HPV) is a risk factor for the development of cervical cancer and, in patients coinfected with HIV, the cervical lesions are more severe and have a worse prognosis [1], [2], [3]. The role of HPV in the pathogenesis of cervical cancer involves interaction of the proteins E6 and E7 of high-risk HPV types with the tumor-suppressor nuclear proteins p53 and pRB, respectively. The protein E7 binds to pRB and displaces the transcription factor E2F, which was originally bound to pRB, thereby increasing the expression of proteins involved in progression to the S phase of the cell cycle. The protein E6 supplements the function of the protein E7 by inactivating p53 and impeding the induction of apoptosis in these cells [4], [5]. Prolongation of the S phase through the action of E6 and E7 may lead to chromosome instability and consequently to alteration of cell ploidy. These events are worsened by the phenomenon of viral integration, which may occur in situations of persistent infection by high-risk HPV, although the transformation phase may occur in stages that precede the viral integration [6], [7], [8], [9].

The DNA index has been used to characterize cell ploidy in cases of endometrial cancer [10], liver cirrhosis [11] and acute lymphoid leukemia [12], and it is important in the prognosis for neoplasia [13]. Some authors have also taken DNA index determinations to be a prospective prognostic parameter for cervical lesions caused by HPV infection [14], [15], i.e. evolution comprising regression, progression or persistence of the cervical lesion [16]. There are no studies on women with HIV-HPV coinfection regarding tissue lesion progression using this technique; and therefore the aim of the present study was to evaluate the association between the DNA index and tissue lesions in patients with HIV-HPV coinfection.

## Methods

### Population and study design

The present study had two stages. In the first stage, comprising a cross-sectional study, the association between the presence of aneuploidy seen by means of flow cytometry and sociodemographic characteristics, habits and characteristics relating to HPV and HIV infection was analyzed. In the second stage, using a cohort design, we compared the frequency of aneuplody in the baseline evaluation with that observed after the follow-up, thus evaluating the evolution of the DNA index.

For the first stage of the study, 409 women were recruited in three referral centers for HIV-AIDS in Pernambuco (Hospital Correia Picanço (HCP), Hospital das Clínicas (HC) and Centro Integrado de Saúde Amaury de Medeiros (CISAM), between May 2007 and August 2011. All the 409 adult women studied were infected with HIV and were attended at the gynecology services of the above mentioned three referral center hospitals for HIV-AIDS. All the women agreed to participate in the study and signed a consent statement.

### Data-gathering and standardization of techniques

A questionnaire for clinical-epidemiological evaluation was applied to all the women who agreed to participate in the study. Following this, cytological examination and colposcopy were performed. For the cytopathological analysis and for the molecular analysis on HPV, cervical scrapings were obtained using cytobrushes while performing the colposcopic examination. To classify the cytological findings, the Bethesda nomenclature was used [17]. The colposcopic findings were classified taking into consideration the classification criteria of the International Colposcopic Nomenclature of Rome [18]. In cases in which colposcopic alterations were observed, a cervical biopsy was performed and the ensuing cervical tissue fragments were sent in 10% formol for histopathological analysis, taking into consideration the Schneider criteria [19].

The cervical scrapings were sent to the Molecular Biology Laboratory of the Pediatric Oncology Service of Instituto de Medicina Integral Prof. Fernando Figueira. The samples were subdivided: one fraction was used to determine the DNA index, while the others were stored at −20°C for subsequent molecular analysis, which included investigating the HPV genome and, in positive cases, molecular typing as described below. The molecular biology laboratory personnel were blinded to the cytological and colposcopic findings.

In the second stage 219 women (53.5%), with a minimum of two visits to the referral centers, were considered. In return consultation, all women were submitted to new cytological examination and colposcopy, with cervical scrapes sent to molecular laboratory for detection of persistence of HPV infection and DNA index determination. No women had done surgical intervention between the clinical evaluations. The time interval between two consecutive visits varied among women.

### HPV detection and typing

Genomic DNA from the cervical samples was extracted using the proteinase K [Invitrogen, CA, USA] protocol and subjected to PCR using the degenerate primers MY09 and MY11 for amplification of the HPV L1 gene as previously described [20]. The PCR product was purified by conventional phenol-chloroform procedure prior sequencing using the Big Dye protocol (Applied Biosystems, SP, Brazil), in accordance with the manufacturer’s description. The chromatograms were firstly viewed using the Mega 5.0 software [21] in order to evaluate the quality of the sequence and comparisons were made with the reference sequences deposited in the GenBank database (http://www.ncbi.nlm.nih.gov), for viral typing identification. The viral isolates were classified in low-risk (types 6, 11, 61, 54, 62, 71, 72, 81, 84, 85, 86) or high-risk (types 16, 18, 31, 33, 45, 52, 53, 56, 58, 59, 66, 69, 70, 82) [22], according to their known capacity in developing cancer.

### Determination of the DNA index

The cervical cells were subjected to cellular lyses using Pharm Lyse lysing buffer (Becton Dickinson, New Jersey, USA) for 30 minutes. After a quick wash, the cells were incubated in 0.5 mL RNA solution (100 µg/mL) supplemented with propidium iodide, a fluorescent reagent that intercalates DNA bases. The DNA fluorescence was measured using flow cytometry, through laser excitation at 488 nm and reading above 600 nm; and DNA index was determined by comparison with labeled normal diploid cells using the ModFitLT V3.0 software (Verity Software House Inc., Topsham, USA). The presence of two peaks on a histogram with an index greater than 1.16 (hyperploidy) or less than 1.00 (hypoploidy), each with more than 10% of the cell population analyzed in the area corresponding to G0–G1 of the cell cycle, was considered to be aneuploidy [15].

### Definition and categorization of variables

The median age of the women studied was 35 years and it was used as the cut-off point to categorize this variable. It coincided with the median found in several studies of risk factors for HPV infection, thus enabling comparison with these studies [23]. The relationship with smoking was analyzed taking smokers and former smokers both separately and as a group, in comparison with nonsmokers. For the variable of alcohol abuse, the term “light drinker” was used to mean women whose frequency of alcohol intake over the preceding three months did not exceed 14 days per month and whose daily dosage was limited to one glass of wine or distilled drink (250 mL), or one can of beer (350 mL); women with greater alcohol intake were categorized as “heavy drinkers”. Drug users were considered to be individuals who were using or had used any illicit drug (smoked, sniffed or injected). The cutoff point for CD4+ T lymphocytes of 200 cells/mm^3^ was used as an expression of the patients’ immunosuppression.

Regarding the evolution of DNA index, “ID Progression” was considered when the first patient sample was diploidy and subsequent samples were aneuploidy, while “ID Regression” means the opposite with first sample being aneuploid and the followed-up sample diploid. “ID persistence” means that cell ploidy did not changed along consecutive cervical scrapes evaluation.

### Statistical analysis

For the statistical analysis, the Stata 10.0 software was used [Stata-Corp LP, College Station, TX, USA]. In the first stage, logistic regression was used to analyse the association between aneuploidy and sociodemographic characteristics, habits and characteristics relating to HPV and HIV infection. In the univariate analysis, odds ratios (OR) with their respective 95% confidence intervals and p values (chi-square or likelihood ratio test) were estimated. The criterion used for inclusion of a variable in the multivariate logistic model was a p value<0.25 in the univariate analysis. For variables to remain in the model, the criterion was a p value<0.05 or an adjustment of the OR value of another variable that was greater than 10%. The Mann-Whitney test was used to analyze the medians of continuous values of unpaired groups. In the second stage the kappa coefficient was used to measure the agreement between the presence of aneuploidy in the baseline evaluation and after the follow-up. Associations producing p<0.05 were considered to be statistically significant.

### Ethical issues

This study was approved by the Research Ethics Committee of Centro Integrado de Saúde Amaury de Medeiros (CEP-CISAM, #0011.0.250.000-05), and all the women invited to participate gave their formal consent.

## Results

### Identification of risk factors for development of aneuploidy in HIV-positive women

The DNA index of cervical cells was measured in 617 samples from 409 HIV-positive women of median age 34 years who were attended at three HIV-AIDS referral centers in Pernambuco. The clinical-epidemiological and laboratory characteristics of the 320 women who completely filled out their research questionnaires, corresponding to 78.2% of the study population, are shown in Table 1. Among the 409 women, 219 returned to the hospital service at least once, for clinical and laboratory follow-up.

**Table 1:** Univariate analysis of the association between DNA index and socio-demographic variables, habits, characteristics associated with HPV infection and characteristics associated with HIV infection among HIV-positive women attending three reference centers for HIV/AIDS in Recife, Brazil, 2008–2010.

Characteristic	Aneuploidy (%)	Diploidy (%)	OR (95%-CI)	*p*
**Socio-demographic characteristics**				
Age (median: 34 years) 0,609				
≥35 years	29(46,77)	129(50,39)	0,86(0,49–1,50)	
<35 years	33(53,23)	127(49,61)	1,00	
Total	62(100)	256(100)		
Illiteracy				0,501
No	6(9,68)	33(12,79)	0,73(0,29–1,82)	
Yes	56(90,32)	225(87,21)	1,00	
Total	62(100)	258(100)		
Schooling				0,758
>8 years	19(30,65)	84(32,68)	0,91(0,49–1,65)	
≤8 years	43(69,35)	173(67,32)	1,00	
Total	62(100)	257(100)		
Income				0,066
<1 minimum wage	16(30,19)	99(44,00)	0,55(0,28–1,04)	
≥1minimum wage	37(69,81)	126(56,00)	1,00	
Total	53(100)	225(100)		
**Habits**				
Smoking				0,648
Smokers	12(22,64)	46(20,44)	1,03(0,49–2,18)	0,928
Former smokers	10(18,87)	56(24,89)	0,70(0,32–1,54)	0,386
Non-smokers	31(58,49)	123(54,67)	1,00	
Total	53(100)	225(100)		
Alcohol intake				0,416
Heavy drinker	4(6,56)	29(11,42)	0,48(0,15–1,49)	0,208
Light drinker	31(50,82)	134(52,76)	0,80(0,45–1,45)	0,480
Abstainer	26(42,62)	91(35,83)	1,00	
Total	61(100)	254(100)		
Smoked drug use				0,535
Yes	10(16,39)	34(13,33)	1,27(0,59–2,74)	
No	51(83,61)	221(86,67)	1,00	
Total	61(100)	255(100)		
Sniffed drug use				0,903
Yes	6(10,00)	24(9,49)	1,06(0,41–2,72)	
No	54(90,00)	229(90,51)	1,00	
Total	60(100)	253(100)		
Drug use				0,796
Yes	10(17,24)	39(15,85)	1,10(0,51–2,37)	
No	48(82,76)	207(84,15)	1,00	
Total	58(100)	246(100)		
**Characteristics associated with HPV infection**				
Number of sexual partners				0,135
≥4 partners	18(31,03)	101(41,74)	0,62(0,34–1,15)	
1–3 partners	40(68,97)	141(58,26)	1,00	
Total	58(100)	242(100)		
Pregnancy on HPV diagnosis 0,334				
Yes	7(11,48)	41(16,47)	0,65(0,27–1,54)	
No	54(88,52)	208(83,53)	1,00	
Total	61(100)	249(100)		
Presence of HPV				0,223
Yes	28(47,46)	95(38,78)	1,42(0,80–2,52)	
No	31(52,54)	150(61,22)	1,00	
Total	59(100)	245(100)		
**Characteristics associated with HIV infection**				
CD4+ T lymphocyte count (*) 0,146				
<200/mm^3^	4(7,69)	34(15,45)	0,45(0,15–1,34)	
≥200/mm^3^	48(92,31)	186(84,55)	1,00	
Total	52(100)	220(100)		
Length of time since HIV diagnosis 0,174				
≥24 months	36(61,02)	128(51,20)	1,49(0,83–2,66)	
<24 months	23(38,98)	122(48,80)	1,00	
Total	59(100)	250(100)		
Use of TARV				0,006
No	20(37,04)	44(19,64)	2,40(1,26–4,57)	
Yes	34(62,96)	180(80,36)	1,00	
Total	54(100)	224(100)		

(*)On the CD4+ T cells count, the result closest to the date of the interview was considered.

### Factors associated with changes to the DNA index

The demographic characteristics of age, schooling level and income level and the habits of smoking, alcohol intake and drug use were similar between the HIV-positive women who presented aneuploidy and diploidy in their cervical cells (Table 1), with income presenting a threshold p value.

HPV infection was present in 47.5% of the aneuploid cervical samples and in 38.8% of the diploid samples from different women with no statistic differences (p = 0.22). Also infection with low-risk HPV (p = 0.43) or high-risk HPV (p = 0.17) did not show any association with alteration of ploidy in the cervical cells. The majority of samples (99%) presented hyperdiploid, i.e., DNA index higher than 1.16.

Most of the women presented CD4+ T lymphocyte counts greater than 200/mm^3^, in both the diploid and the aneuploid group. The length of time with a diagnosis of HIV did not present any association with aneuploidy but, on the other hand, aneuploidy was shown to be associated with use of antiretroviral treatment (p = 0.006) (Table 1).

After the multivariate analysis, use of antiretroviral treatment (p = 0.001) and income (p = 0.007) remained in the final model.

### Ploidy and alteration of cervical cells

Aneuploidy was observed in a total of 127 women over the study period. Of these, 12 provided follow-up samples, thus making a total of 139 samples with aneuploidy. Among the women with oncotic cytology results, the DNA index was measured in the baseline evaluation of this study in 56 women with aneuploidy. Of these, 12 returned more than once to the hospital service. Another 46 women with aneuploidy did not fill out the research questionnaire. In total, 114 aneuploid samples with oncotic cytology results were obtained.

Out of the 19 aneuploid samples (16.7%) showing cytological alterations, six (31.6%) were diagnosed with atypical squamous cells of undetermined significance (ASCUS), 10 (52.6%) with low-grade squamous intraepithelial lesions (LSIL) and three (15.8%) high-grade squamous intraepithelial lesions (HSIL). Out of the 45 diploid cells showing cytological alterations, 11 (24.5%) were classified as ASCUS, one (2.2%) as atypical squamous cells that did not allow high-grade lesions to be ruled out (ASC-H), two (4.5%) as presence of HPV, 24 (53.3%) as LSIL and seven (15.5%) as HSIL. Considering the 531 cervical samples that were evaluated regarding the DNA index and cytological alterations, ploidy was not associated with the presence of cytological alterations (p = 0.348) (Table 2).

**Table 2:** Analysis of cell ploidy in accordance with cervical intraepithelial lesions in HIV-positive women attending three reference centers for HIV/AIDS in Recife, Brazil, 2008–2010.

Cytological alterations	Aneuploidy (%)	Diploidy (%)
ASCUS	6(31,6)	11(24,5)
ASC-H	0	1(2,2)
HPV	0	2(4,5)
LSIL	10(52,6)	24(53,3)
HSIL	3(15,8)	7(15,5)
Total	19	45

In an exploratory manner, the association between presence of aneuploidy and histological lesions was evaluated among women with an indication for biopsy by means of colposcopy. Considering only the 54 diploid samples with biopsy results, it was found that CIN was present in 31.5% of the samples, while in the 15 aneuploid samples with histological evaluation, 66.7% showed CIN, thus demonstrating am association between histological lesions and detection of aneuploidy through the DNA index (p = 0.030) (Table 3).

**Table 3:** Association between ploidy of cervical cells and cervical biopsy results in HIV positive women attending three reference centers for HIV/AIDS in Recife-PE, 2008–2010.

Histology	Aneuploidy (%)	Diploidy (%)	*P*
Presence of CIN	10 (66,7)	17 (31,5)	0,030
Absence of CIN	5 (33,3)	37 (68,5)	
Total	15 (100)	54 (100)	

Considering the 69 samples that had biopsy and DNA index results, it was found that in determining the cell ploidy, the specificity was 88.1% (73.6–95.5%) and the sensitivity was 37% (20.1–57.5%) for CIN in HIV-positive women.

### Evolution of the DNA index and HPV infection in HIV-positive women

Out of the 409 women studied, 219 (53.5%) returned to the hospital service at least once, for follow-up over different time intervals ranging from 6 to 48 months, among whom 92 (42.2%) returned between 13 and 24 months after the first consultation and 69 (32.3%) between 6 and 12 months afterwards. The evolution of the cervical lesion regarding the DNA index was analyzed in relation to 178 women. The 41 women for whom information on the DNA index was only available from a single occasion were not included in this analysis. Among the 178 samples, information on the DNA index from cervical cells was available from three or more occasions.

Out of the 40 women with aneuploid samples at the first evaluation, 31 (77.5%) regressed to diploidy by the end of the follow-up period. Considering the 137 women with diploidy at the first evaluation, 105 (76.6%) remained diploid and 32 (23.4%) progressed to aneuploidy. Kappa measurements were used to evaluate the concordance of aneuploidy detection between the initial sample and the last sample analyzed. This showed weak concordance (k = 0.031), according to the kappa interpretation table proposed by Landis and Koch (1977) [24] (Table 4).

**Table 4:** Persistence of aneuploidy in HIV-positive women attending three reference centers for HIV/AIDS in Recife-PE, 2008–2010.

	Final evaluation		Agreement	
Initial evaluation	Positive	Negative	Expected	Kappa
**Aneuploidy**				
Positive	9	31	0,64	0,031
Negative	32	105		
Total	42	136		

Among the 33 women with progression to aneuploidy, 17 had oncotic cytology results from their samples at diagnosis and at the return consultation. Among these 17 women, 13 did not present any cytological alteration at either of the two times evaluated; cytological regression was observed in two women who had diagnoses of LSIL and HSIL at the initial assessment and became negative; persistence of cytological alterations independent of the lesion grade was found in one woman with an initial diagnosis of ASC-H and a diagnosis of HSIL at the return consultation; and progression was identified in only one woman who had a negative sample at the beginning of the study, followed by a sample with ASCUS.

Out of the 105 women with a diploid DNA index on both occasions, the median level of CD4+ T cells in peripheral blood was 455/mm^3^, which was similar to the median CD4+ level of 461/mm^3^ presented by the nine women with an aneuploid DNA index on both occasions (p = 0.205).

## Discussion

### DNA index, cervical lesion and HPV

The frequency of aneuploidy in cervical cells was greater in the HIV-positive women who were not using antiretroviral treatment than in those who were doing so. Although an association was found between the presence of cervical tissue lesions and the DNA index, the latter was not predictive of the evolution of the cervical lesion, thus suggesting that the progression of the cervical lesion to cancer in HIV-positive women may also be altered through the improvement in immunological state that is provided by use of antiretroviral therapy.

In the present study, no association was observed between aneuploidy and cytological alterations or HPV detection. Although there was an association between aneuploidy and the presence of cervical tissue lesions, the DNA index obtained through flow cytometry was not predictive of the evolution of the cervical lesion.

Furthermore, in the present study, higher income had a protective effect against development of aneuploidy. It is possible that the income level was measuring another factor that was not evaluated in this study.

Aneuploidy is frequently found in severe cervical lesions in HIV-negative women [25], [26], but this was not observed in the present study in relation to oncotic cytology, perhaps because of the low frequency of cases of altered cytology (12.0%). Oncotic cytological analysis is an important low-cost test for screening for cervical lesions, but it is less specific that histological analysis for characterizing precancerous lesions, in which higher frequency of aneuploidy would be expected [27].

The presence of high-risk HPV contributes towards cervical lesions, especially CIN [2], [28], [29]. High-risk HPV types are closely associated with appearance and persistence of pre-neoplastic cervical lesions [1], [2], [30], given that the viral proteins E6 and E7 bind to and inhibit the action of the human proteins p53 and pRB, which control the cell cycle. This culminates in establishment of chromosomal aberrations and, consequently, appearance of aneuploidy [5], [31]. Although this relationship is known, no association between aneuploidy and HPV infection was found in the present study, perhaps because of the preserved immunological state of the HIV-positive women in this study, who did not present cytological alterations, were mostly using antiretrovirals and had normal CD4+ T lymphocyte counts. However, it was observed that non use of antiretrovirals was associated with the presence of aneuploidy (p = 0.001). It is known that chronic vaginal infections caused by anaerobic bacteria act as cofactors for CIN to appear, with consequent change of cell ploidy, independent of HIV and HPV infection [32]. In the present study, no data on associated bacteriosis and other infections of the female genital tract were evaluated, thereby limiting the understanding of this issue.

HIV infection may in itself increase the risks of cytological alterations and HPV infection independently [33]. In this regard, it is expected that after use of antiretrovirals starts, there will be exacerbation of tissue inflammation caused by silent opportunistic cervical infections that are revealed through the reconstitutive immunotherapy [34], [35]. An association between antiretroviral use and protection against emergence of CIN was recently demonstrated in HIV-positive women [36].

**Figure 1:**
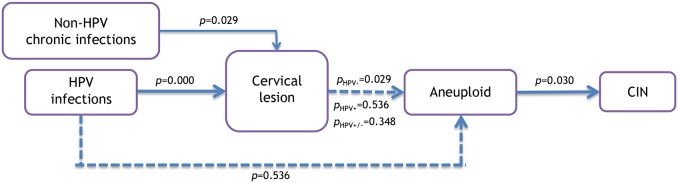
Model of progression of cervical lesions to cervical cancer in HIV-positive women treated with HAART.

Our data suggest that the model for cervical lesion progression to cancer in HIV-positive women may be altered through the improvement of their immunological state that is provided by antiretroviral use and clinical follow-up (Figure 1). In the proposed model, occurrences of CIN are associated with the presence of aneuploidy (p<0.030), but aneuploidy is not associated with the presence of cytological alterations (p = 0.348), or with HPV infection of low grade (p = 0.43) or high grade (p = 0.17) in HIV positive patients; on the other hand, in HIV-negative patients were observed association between aneuploidy and cervical lesion. If cervical lesions are taken to be an expression of cervical HPV infection or chronic infections [28], [29], [32], while knowing that HPV may be present in the absence of cervical lesions (depending on the patient’s immunological state), the progression of the disease among HIV-positive patients will be influenced by their adherence to antiretroviral treatment.

### Evolution of cervical lesions with regard to aneuploidy

In relation to the DNA index, the HIV-positive women showed a progression rate (23.9%) that was lower than the regression rate (77.5%), with a low frequency of cytological alterations (12.0%). In the literature investigated, no other study using the DNA index to evaluate the evolution of cervical lesions in HIV-positive women was found. However, it is known that in HIV-negative women, the aneuploidy rate has a relationship with the grade of the cytological lesion at diagnosis, and that the greater the severity of the cytological lesions in aneuploid samples is at diagnosis, the greater the likelihood will be that the cervical lesions will persist or progress in follow-up samples from these patients [25], [26].

The presence of changes in ploidy in cervical cells alone was shown to have low sensitivity (37%) and specificity (88%) for detecting CIN. However, it has been reported that a combination of HSIL cytomorphology, presence of oncogenic HPV and aneuploidy presented 100% sensitivity and 91.3% specificity for detecting lesions that progress to severe cervical lesions (CIN II+) [26].

Recently, it was demonstrated that changes to the ploidy of cervical cells can be identified through flow cytometry, using five different parameters relating to the cell cycle, with sensitivity and specificity of 100% [30]. In the same study, aneuploidy alone was capable of detecting cervical cancer with sensitivity of 83.9% (72.3–92.0) and specificity of 94.7% (82.2–99.2). The main differences between the present study and the study by Chhavi et al. were that the latter was a case-control study on HIV-negative Indian women in which 62% of the women presented cervical cancer (cases) and 38% of the women had been hysterectomized due to benign causes (controls), while the present study was a cohort study on HIV-positive Brazilian women with low prevalence of cytological abnormalities and thus with few indications for performing biopsies. This suggests that there is a difference in the sensitivity of the method in populations with differences in the grade of the cervical lesion.

In summary, it can be concluded that the presence of aneuploidy has a relationship with occurrences of precancerous cervical lesions. This study showed that the frequency of aneuploidy was low among women with CIN (37%), but in women with aneuploidy, the frequency of CIN was high (66%).

Although an association was found between the presence of cervical tissue lesions (CIN) and determination of the DNA index through flow cytometry, the latter was not predictive of the evolution of the cervical lesion.

There was a significant loss of data, given that the return rate was relatively low (47.5%). This may have limited the exploitation of the DNA index technique for detecting cervical lesions and evaluating their evolution among HIV-positive women.

In most of the sample, no colposcopic atypias that could have been indicative for performing biopsies were observed. It is possible that another study with a larger number of women with colposcopic and/or cytological alterations might contribute towards better comprehension of the association between the DNA index and the presence and evolution of cervical lesions in HIV-positive women.
